# ‘Unemat Rubi’, a new spineless pineapple cultivar and resistant to fusariosis for the international market

**DOI:** 10.1038/s41598-025-00971-x

**Published:** 2025-05-13

**Authors:** Dayane Castro Silva, Willian Krause, Rayla Nemis de Souza, Eileen Azevedo Santos, Dejânia Vieira de Araújo, Celice Alexandre Silva, Aline Vidor Melão Duarte, Wandreilla Moreira Garcia, Leonarda Grilo Neves

**Affiliations:** 1 Plant Molecular Genetics and Phytopathogens Laboratory, Mato Grosso State University, Tangará da Serra, 287 Brazil; 2 Phytopathology Laboratory, Mato Grosso State University, Tangará da Serra, 287 Brazil; 3 Botany Laboratory, Mato Grosso State University, Tangará da Serra, 287 Brazil; 4 Estácio do Pantanal University Center, Cáceres, Brazil; 5 Laboratory of Plant Genetic Improvement, Mato Grosso State University, Cáceres, Brazil

**Keywords:** *Ananas comosus* var. *comosus*, Plant breeding, *Fusarium guttiforme*, Correlation networks, Canonical variables, Genetic engineering, Genetics

## Abstract

Pineapple cultivation worldwide depends on a limited number of cultivars. In Brazil, the cultivar ‘‘Pérola’’ accounts for 85% of the commercial planted area but is susceptible to fusariosis, the most significant disease affecting pineapple crops. This study introduces the cultivar ‘Unemat Rubi’, emphasizing its superior fruit quality and resistance to fusariosis through multivariate analyses, correlation networks, and genetic parameters. Eighteen genotypes were evaluated for qualitative and quantitative traits and resistance to fusariosis, using a randomized block design with five replications and 20 plants per plot. The genotypes were grouped into two main clusters based on their resistance or susceptibility to fusariosis. ‘Unemat Rubi’ was classified in Cluster I, along with its female parent (‘BRS Imperial’), sharing resistance to fusariosis, cylindrical fruits with yellow pulp, and no leaf spines. However, ‘Unemat Rubi’ was superior to ‘BRS Imperial’ in terms of fruit weight and diameter, presenting a mass above 1.5 kg and an average diameter above 10 cm. No correlations were observed between the groups of chemical and physical traits of fruit and resistance to fusariosis, only between the groups of physical and chemical traits. There was a high and positive correlation for FMWOC and FMWC (0.99) and a strong and significant correlation between DLL with FMWOC and FMWC, both with 0.74. Heritability estimates exceeded 90% for most traits, except for fruit diameter. The cultivar ‘Unemat Rubi’, registered at the Brazilian Ministry of Agriculture and Livestock under number 56,622, represents a significant advancement in pineapple breeding by integrating superior fruit quality with fusariosis resistance, making it a promising candidate for commercial expansion.

**Supplementary Information:**

The online version contains supplementary material available at 10.1038/s41598-025-00971-x.

## Introduction

Pineapple (*Ananas comosus* var. *comosus*) is the most economically significant member of the family Bromeliaceae. It is grown extensively in tropical and subtropical regions, ranking second in the global production of tropical fruits, after bananas^1^. Indonesia is the world’s largest pineapple producer, followed by Philippines, Costa Rica, China and Brazil. Pineapple production in Brazil reached 2.39 tonnes in 2023, on an area of 63,943 ha, with a mean yield of 24,891 kg ha^− 1^^2^. The most widely grown pineapple cultivars worldwide are MD-2, ‘Smooth Cayenne’, ‘Singapore Canning’, ‘Queen’, ‘Española Roja’, ‘Pérola’, and ‘Manzana’^3^.

‘Pérola’ is the most widely consumed pineapple cultivar in the fresh fruit market in Brazil, representing more than 85% of the commercial planted area^3^. However, its white pulp limits its acceptance in the international market, where yellow-pulp pineapples are strongly preferred. Additionally, ‘Pérola’ has other disadvantages, including spines on its leaves and susceptibility to fusariosis, the most significant disease affecting pineapple crops in Brazil. The primary pathogen responsible for this disease in pineapple plants is Fusarium guttiforme, although other species have also been reported^4,5^.

Fusariosis severely decreases crop yields and the commercial value of the fruits, causing yield losses that may reach up to 40% of marketable fruits and 20% of planting material in some regions and many pineapple fields over the country^6^. The control of fusariosis is carried out using healthy planting material, eradication of infected plants during the vegetative plant cycle and application of fungicides from the appearance of the inflorescence until the closing of the last flowers. However, the most economical and environmentally safe strategy is the use of resistant cultivars^7^.

Screening methods for resistance to *Fusarium guttiforme* in breeding programmes are carried out based on pathogen inoculation in seedlings and molecular-level genetic improvement, which involves the identification and manipulation of genes that confer resistance to this fungal disease. However, the most commonly used method is the inoculation of the fungus in the seedlings.

Pineapple breeding programs in Brazil have prioritized the development of spineless cultivars resistant to fusariosis in response to marketing demands, while incorporating other desirable traits, including rapid plant growth, yellow pulp, cylindrical fruit shape, high pulp sugar content, low pulp acidity, and improved resistance to internal browning during transport and storage^8,9^.

There are 24 cultivars registered as of 2024 between fruit and ornamental plants. Among the fruit cultivars, these include ‘BRS Imperial’^10^, ‘BRS Ajubá’^11^ and ‘BRS Vitória’^6^, developed by the Brazilian Agricultural Research Corporation (EMBRAPA); ‘IAC Fantástico’^12^, developed by the Agronomic Institute of Campinas (IAC); and ‘Unemat Esmeralda’, developed by the Mato Grosso State University (UNEMAT) breeding program. ‘BRS Imperial’, ‘BRS Ajubá’, ‘BRS Vitória’, and ‘IAC Fantástico’ are resistant to fusariosis but have limitations, such as small fruit size. Although Unemat Esmeralda is resistant to fusariosis, its white pulp does not meet the requirements of the international market.

The development of pineapple cultivars resistant to *F. guttiforme*, while incorporating a greater number of advantageous traits for both growers and consumers, requires understanding, exploring, and effectively managing genetic variability within a well-structured breeding program, which relies on the characterization and evaluation of germplasm. In this context, a thorough assessment of plant genetic resources plays a fundamental role in guiding breeding decisions. Determining the traits of plant genetic resources is therefore crucial for developing effective breeding strategies^13^.

Typically, pineapple breeding programs begin by obtaining segregating populations from crosses between resistant and susceptible cultivars. These genotypes are then evaluated for agronomically important traits, such as fruit weight without crown and soluble solids, across several cycles of vegetative propagation. Superior individuals selected during this phase are subsequently inoculated with *F. guttiforme* to assess their responses to the pathogen^14^.

The extensive land area of Brazil and its diverse edaphoclimatic conditions significantly intensify the genotype × environment interaction, requiring the development of cultivars adapted to various production regions. In this context, UNEMAT launched a pineapple breeding program in 2012 aimed at developing new cultivars. To achieve this, a germplasm bank was established, containing nine accessions that were subsequently characterized and evaluated for morphoagronomic traits and disease resistance^15,16^. Several crosses were then conducted, leading to the selection of eight superior clones, including ‘Unemat Rubi’, which was registered as a cultivar at the Brazilian Ministry of Agriculture and Livestock (MAPA) under number 56,622^17,18^.

Therefore, this study introduces the cultivar ‘Unemat Rubi’, providing a comparative analysis with other pineapple cultivars and clones regarding fruit quality attributes and fusariosis resistance, based on multivariate techniques, correlation networks, and genetic parameters.

## Materials and methods

### Experimental area

The experiment was conducted at the experimental area of the Mato Grosso State University (UNEMAT), in Tangará da Serra, Mato Grosso, Brazil (14° 39’ S, 57° 25’ W, and 321 m altitude). The soil of the experimental area was classified as a Latossolo Vermelho distroférrico of clayey texture, according to the Brazilian Soil Classification System^19^. The region’s climate was classified as Aw, tropical, exhibiting two well-defined seasons: a rainy summer and a dry winter^20,21^. The mean annual rainfall depth is 1,800 mm, with the rainiest period from November to March, and the mean temperature is approximately 25 °C^22^.

### Genetic material and experimental design

Ten superior clones that were selected in previous selection cycles were evaluated^18^ and eight cultivars were evaluated, six of which were used as parents in the genetic improvement programme of the pineapple plant at Unemat. The parental cultivars were selected due to the availability of only six cultivars in the germplasm bank for crosses. The cultivars were acquired through donations from commercial producers in the State of Mato Grosso, except for the cultivar ‘Unemat Rubi’, which was developed at Unemat in Tangará da Serra—MT (Table 1).

**Table 1:** Origin of the cultivars acquired from small properties in the state of Mato Grosso for the establishment of the active germplasm bank (BAG) at Unemat, Tangará Da Serra campus.

Cultivar	Municipality of origin
‘Pérola’	Tangará da Serra—MT
‘Jupi’	Terra Nova do Norte—MT
‘Smooth Cayenne’	Terra Nova do Norte—MT
‘BRS Ajubá’	Tangará da Serra—MT
‘BRS Imperial’	Tangará da Serra—MT
‘BRS Vitória’	Unemat Tissue Culture Laboratory—Tangará da Serra—MT
‘IAC Fantástico’	Unemat Tissue Culture Laboratory—Tangará da Serra—MT
‘Unemat Rubi’	Unemat—Tangará da Serra—MT

**Fig. 1:**
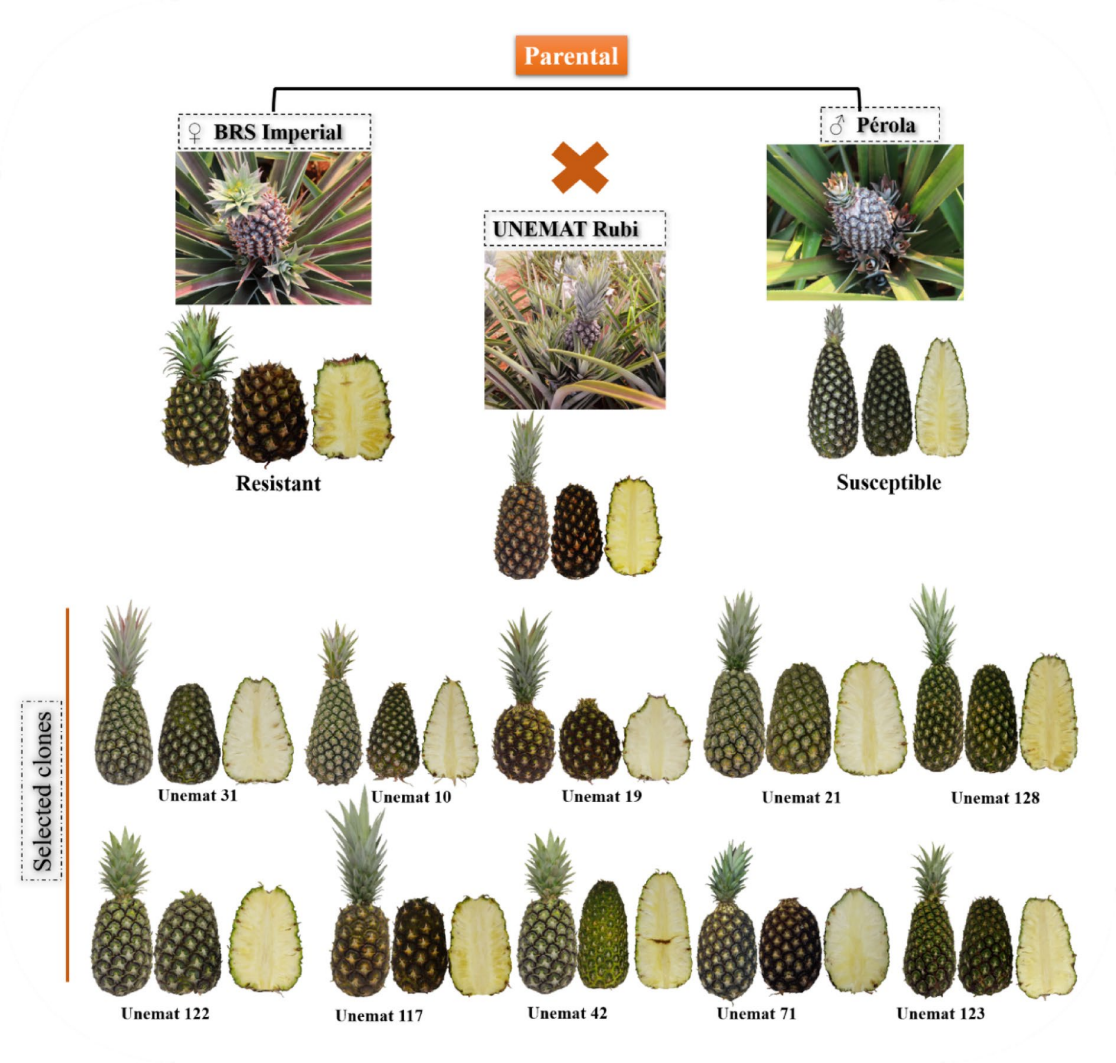
Plant and fruit morphological traits of ‘Unemat Rubi’, ‘BRS Imperial’ (female parent), and ‘Pérola’ (male parent), and fruit aspects of the other clones evaluated in the experiment. Tangará da Serra, MT, Brazil, 2020.

These pineapple clones were generated through hybridizations between cultivars (‘IAC Fantástico’ × ‘Jupi’, ‘BRS Imperial’ × ‘Pérola’, ‘BRS Imperial’ × ‘Smooth Cayenne’, and ‘BRS Vitória’ × ‘Smooth Cayenne’) and selected for fruit quality-related agronomic traits using the Restricted Maximum Likelihood/Best Linear Unbiased Prediction (REML/BLUP) methodology^18^ (Table 2; Fig. 1). The experiment was conducted in a randomized block design, with five replications and 20 plants per plot.

**Table 2:** Number of pineapple genotypes generated from four crosses between resistant and susceptible cultivars to fusariosis. Tangará Da Serra, Mato Grosso, Brazil, 2020.

Parents		Identification of genotypes
Female (resistant)	Male (susceptible)	
‘BRS Imperial’	‘Smooth Cayenne’	10, 19, and 21
‘BRS Vitória’	‘Smooth Cayenne’	31 and 42
‘IAC Fantástico’	‘Jupi’	71
‘BRS Imperial’	‘Pérola’	‘Unemat Rubi’, 117, 122, 123, and 128
Total		11

The crosses were conducted in the morning, between 06:00 and 09:00 a.m. Emasculation of flowers from the female parents was not needed due to the high incompatibility of the plants. Cross-pollination was performed by collecting flowers from the male parents, using tweezers, and placing them on Petri dishes. The anthers were then detached to pollinize the flowers of the female parent (‘BRS Imperial’). Following pollination, the flowers were covered with paper bags to prevent pollination by insects.

The resulting fruits were harvested 60 days after crossing, and seeds were collected and disinfected in 70% alcohol and sodium hypochlorite for 15 min. Subsequently, these seeds were germinated in 250-mL transparent plastic boxes (Gerbox) with autoclaved sand, which were stored in a growth chamber at 24 °C with 12-h photoperiod and irrigated three times a day with sterile distilled water. Plantlets were transplanted into trays 60 days after germination and left in a greenhouse covered with a 70% shade screen. Subsequently, seedlings were transplanted to beds inside a greenhouse covered with a 50% shade screen.

### Planting and conduction of the experiment

Clones and cultivars were planted in July 2022 using sucker-type seedlings with sizes ranging from 25 to 35 cm. The planting spacing was 1.20 × 0.40 × 0.40 m (double rows), with a density of 31,250 plants ha^− 1^. Lime and soil fertilizers were applied as basal dressing and as topdressing based on the soil analysis^23^. The soil analysis results for the 0–20 cm layer were: pH (water) of 5.2; 1.48 mg dm^− 3^ of P; 80 mg dm^− 3^ of K; 0.12 cmolc dm^− 3^ of Al; 0.85 cmolc dm^− 3^ of Ca; 0.29 cmolc dm^− 3^ of Mg; 3.75 cmolc dm^− 3^ of H + Al; sum of bases of 1.3 cmolc dm^− 3^; cation exchange capacity (pH 7.0) of 5.05; 25.75% base saturation; and 12 g dm^− 3^ of organic matter. Irrigation was carried out according to the recommendations for the crop according to Pérez et al.^24^.

Artificial floral induction was performed 12 months after planting by applying an ethepon solution to the center of the plant apex in the late afternoon, as recommended for pineapple cultivation^25^. Weed control was initially performed by hand weeding all clones and cultivars. Subsequently, chemical weeding was used with the herbicide Krovar (diuron + bromacil) at a dosage of 4 kg ha^−1^, which allowed efficient weed control throughout the plantation. Fruits were harvested five to six months after artificial flower induction, as recommended for pineapple crops^26^.

### Plant characterization and fruit quality

Vegetative traits were evaluated at the onset of inflorescence development, whereas fruits were evaluated when they were at the green stage^27^. Ten plants per replicate were evaluated with five replicates for each clone and cultivar. The following vegetative traits were assessed: plant height (cm), measured from the ground level to the highest leaf in the plant’s natural position; D-leaf length (cm), measured from its base on the stem to its tip; and number of seedlings (suckers) per plant.

The following fruit traits were assessed: fruit weight (g), with and without the crown, measured using a digital balance; mean fruit diameter, calculated by the mean of the fruit upper, middle, and lower diameters, measured with a ruler; soluble solids (SS; in °Brix), titratable acidity (TA), and SS-to-TA ratio.

Titratable acidity (TA) was determined via titration with 0.1 N sodium hydroxide (NaOH): 10 mL of fruit juice was diluted with distilled water to a total volume of 50 mL; then, 2–3 drops of 1% phenolphthalein indicator were added; the mixture was shaken and titrated with 0.1 N NaOH until it turned slightly pink. The amount of NaOH used was recorded to calculate the percentage of citric acid in the juice^28^ Soluble solids (SS) were measured using a digital refractometer with a scale ranging from 0 to 95% Brix (RTD-95). Then, the maturation index (SS-to-TA ratio), which reflects the ripening progression, was calculated.

Four key qualitative traits were also assessed: fruit shape, pulp color, presence of spines (spinescence) on leaves^27^, and resistance to fusariosis.

### Phenotyping for fusariosis resistance

Commercial cultivars and the clones selected through REML/BLUP were evaluated for resistance to fusariosis. Seedlings with 10 to 15 cm from each genotype were inoculated with a mixture of fungal isolates of *Fusarium guttiform*e. A total of 18 genotypes were evaluated, using a randomized block experimental design with three replications and five plants per plot. Susceptible cultivars (‘Pérola’, ‘Jupi’, and ‘Smooth Cayenne’) and resistant cultivars (‘BRS Imperial’, ‘BRS Vitória’, ‘IAC Fantástico’, and ‘BRS Ajubá’) were used as controls.

Fungal isolates obtained in the region (Tangará da Serra) were purified through through monosporic culture and characterized based on morphological analysis according to Nirenberg and O’Donnell^29^. Three isolates were selected based on a pathogenicity test, these being the most pathogenic, grown individually for 10 days in Petri dishes containing potato dextrose agar (PDA) and maintained at 25 °C with a 12-h photoperiod. Twenty 20 mL of sterile distilled water was added to each plate and scraped using a Drigalski spatula. The suspended material was filtered through sterile gauze and adjusted to a concentration of 10^5^ conidia mL^− 1^ after counting in a Neubauer hemacytometer under an optical microscope^30^. Subsequently, the three cultured isolates were combined in a single container, forming a single suspension^18^.

The inoculation was carried out in May 2022, following the methodology proposed by Souto and Matos^30^, which consists of making three to four holes in the seedling stems with a perforating tool and subsequently submerging them for three minutes in the conidial suspension mixed with isolates. Seedlings treated with sterile distilled water were used as controls. Immediately after inoculation, the seedlings were planted in beds containing sterile clayey-sandy substrate (clay soil and washed sand) in a 3:1 ratio. The response of plants to infection by *F. guttiforme* was evaluated between 90 and 120 days after inoculation^31^.

Disease severity was assessed every 15 days after inoculation using an adapted scoring scale proposed by Santos et al.^32^, ranging from 0 to 5, where 0 = no symptoms; 1 = initial exudation; 2 = mild exudation; 3 = severe exudation; 4 = necrotic basal leaves with initial wilting; and 5 = severe wilting leading to plant death.

The scores of each plot were converted to the disease index (DI) proposed by Mckinney^33^, according to Eq. (1):

1 $$\:\text{DI\:}{=}\:\sum\:\left(\frac{\text{f\:} \times \text{\:v}}{\text{n}} \times {\text{x}}\right) \times {100},$$

where f = number of plants with a given score; v = observed score; n = total number of plants evaluated; x = maximum score in the scale.

The DI values over time for each plot were used to calculate the area under the disease progress curve ($\:\text{A}\text{U}\text{D}\text{P}\text{C}$), using Eq. (2). AUDPC consists of determining the evolution curve of disease symptoms in relation to time, as a result of the effects of the host, pathogen and environment, interacting with each other^34^.

2 $$\:\text{A}\text{U}\text{D}\text{P}\text{C}=\:\sum\:\left[\left(\frac{\left(\text{y}1\:+\:\text{y}2\right)}{2}\right)\times\:\:\left(\text{t}1-\text{t}2\right)\right],$$

where y1 and y2 = two consecutive evaluations at times t1 and t2, respectively^35^.

The pathogen was identified at the end of the experiment through incubation of the evaluated plant material: the structures were observed under an optical microscope to confirm the infection in the genotypes by the pathogen.

**Fig. 2:**
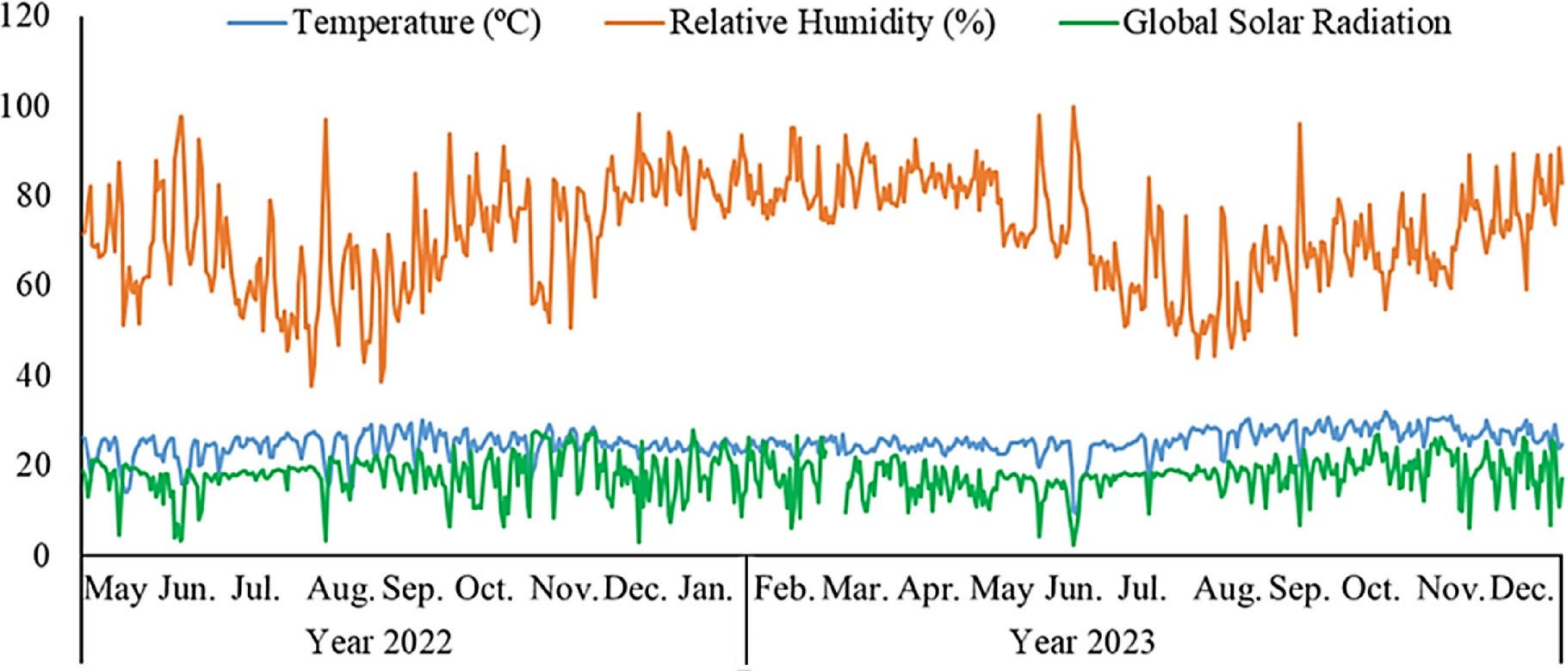
Average air temperature, global solar radiation and relative humidity during the experiment. Tangará da Serra, MT, Brazil.

The meteorological data recorded throughout the experiment, covering the planting period, vegetative analysis of the genotypes, physical-chemical analysis of the fruits and the evaluation of resistance to fusariosis, are presented in Fig. 2^36^.

### Statistical analysis

Estimates of variance components and genetic parameters for fruit traits and AUDPC were obtained using analysis of variance (ANOVA) based on the model $\:\:{Y}_{ijk}=\:\mu\:+\:{B}_{k}+\:{G}_{i}+\:{\epsilon\:}_{ijk}$, where µ is the overall mean, $\:{G}_{i}$ is the random effect of the *i*-th genotype (i = 1.2,…,g), $\:\:{\varvec{B}}_{\varvec{k}}$ is the effect of the *k*-th block (*k* = 1.2,…,*r*), and $\:{\epsilon\:}_{ijk}$ is the experimental error for each observation, assumed to follow *NID* (0,$\:{{\upsigma\:}}^{2}$). Data normality was analyzed using the Shapiro-Wilk test to confirm the assumptions of ANOVA. Following ANOVA for each variable, the following parameters were estimated: phenotypic variance, genetic variance, environmental variance, broad-sense heritability, coefficient of genotypic variation, variation index, and accuracy.

Quantitative and qualitative variables were subjected to cluster analysis using the Gower distance, calculated through the Genes software^37^. The resulting matrix was used for clustering through the unweighted pair group method with arithmetic mean (UPGMA) in the MEGA 11 software^38^. The number of clusters was estimated based on the criterion proposed by Mojena^39^, with cutoffs between 73% and 80% dissimilarity and *k* = 1.25. Each cluster was identified by a color, and subclusters by distinct symbols. Boxplot graphs were generated for the quantitative variables, which were subsequently grouped using the Scott-Knott test (*p* ≤ 0.05) in the Rbio software^40^. Pie charts were created in Excel to display the percentage of individuals in each class corresponding to the qualitative variables.

Canonical variables were developed for physical and chemical fruit traits and resistance to fusariosis (AUDPC), with the mean scores for each combination of factors displayed in a two-dimensional plot using the biplot technique in the Rbio software^40^. The significance of each trait was assessed based on the canonical variable loadings.

Correlation networks were created to assess the interaction of physical and chemical fruit traits with resistance to fusariosis. Analyses were conducted using the R 3.1.2 software^41^, and the correlation networks were constructed using the Qgraph package^42^. The Fruchterman–Reingold^43^ algorithm was applied to generate a force-directed layout for the network, where the proximity between nodes (traits) was proportional to the absolute correlation values between them. Positive and negative correlations were represented by green and red lines, respectively. Correlation values were classified according to Shimakura and Ribeiro Júnior^44^, where, regardless of the sign, a correlation is very weak (0.00 to 0.19), weak (0.20 to 0.39), moderate (0.40 to 0.69), strong (0.70 to 0.89), or very strong (0.90 to 1.00).

## Results and discussion

### Phenotypic characterization of pineapple genotypes using the gower distance

**Fig. 3:**
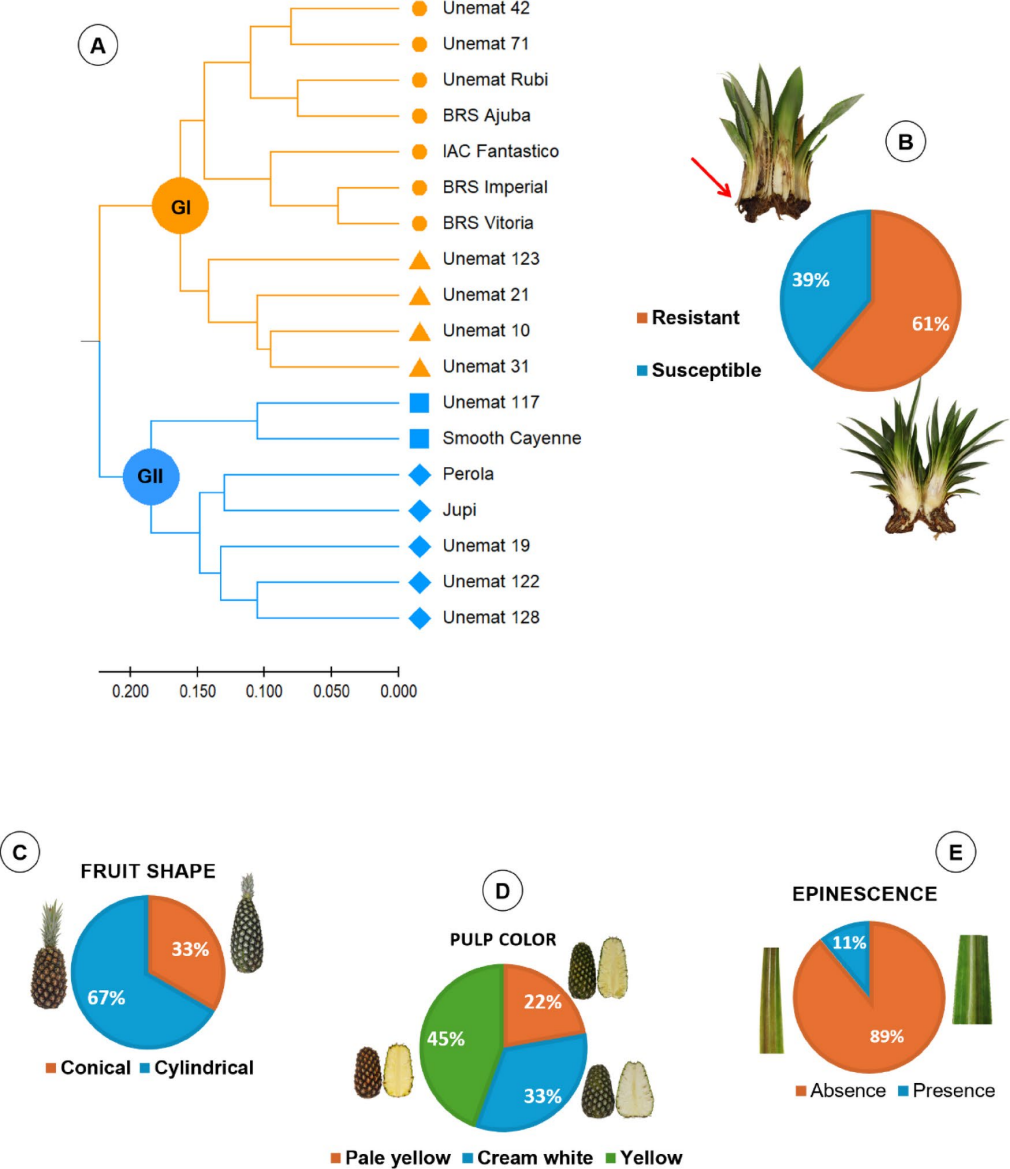
(**A**) Dendrogram from the unweighted pair group method with arithmetic mean (UPGMA) (CCC = 0.71) based on the Gower distance matrix for 10 quantitative traits related to resistance to fusariosis and fruit quality, two multicategorical traits, and two binary traits. (**B**–**E**) Pie charts displaying the percentage of individuals regarding: response to *Fusarium guttiforme*, with a red arrow indicating the presence of stem rot in seedlings of the cultivar ‘Pérola’ (**B**); fruit shape (**C**); pulp color (**D**); and leaf spinescence (**E**).

The genetic dissimilarity among 10 hybrids and eight cultivars was estimated based on quantitative and qualitative variables related to fruit quality and resistance to fusariosis. Regarding qualitative traits, 61% of the evaluated genotypes were resistant to fusariosis, which was evaluated through field trials, 67% exhibited conical fruits, 45% displayed yellow fruit pulp (widely preferred by the international market), and 89% exhibited no leaf spines (Fig. 3).

The evaluated genotypes were grouped into two main clusters using the UPGMA method, which showed the highest cophenetic correlation (0.71). The cutoff points in the dendrogram were determined between 73 and 80% dissimilarity, according to Mojena^39^. This statistical criterion calculates the relative size of the distance levels in the dendrogram without requiring prior information about cluster configuration^45^. The most similar genotypes were the cultivars ‘BRS Imperial’ and BRS Vitoria (0.09), while the most divergent were Unemat 123 and the cultivar ‘Pérola’ (0.66) (Fig. 3).

Cluster I consisted of six clones and five cultivars, all resistant to fusariosis and lacking leaf spines (indicated by orange lines): seven exhibited cylindrical fruits, and seven exhibited yellow pulp. Cluster II consisted of four clones and three cultivars, all susceptible to fusariosis (blue lines): six exhibited cylindrical fruits, three had yellow pulp, and two showed the presence of leaf spines (Fig. 3).

‘Unemat Rubi’ was classified in Cluster I, along with its female parent (‘BRS Imperial’). Both share resistance to *Fusarium guttiforme*, cylindrical fruits with yellow pulp, and the absence of leaf spines. Breeding programs in Brazil and worldwide have prioritized selecting genotypes that produce cylindrical fruits due to their uniform chemical traits and ease of transportation^3^. Moreover, yellow pulp is preferred in the international market for its higher appeal^46^.

**Fig. 4:**
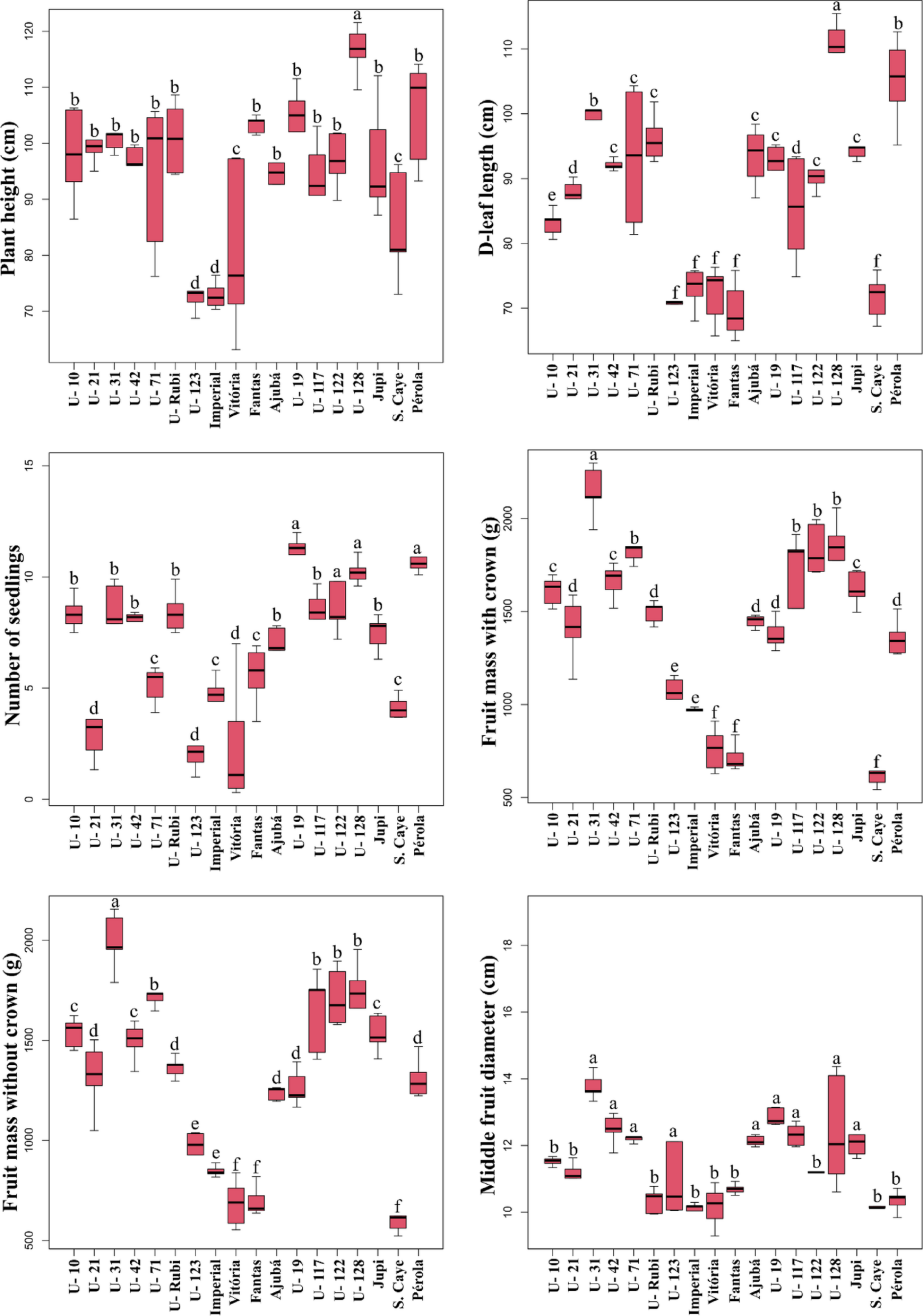

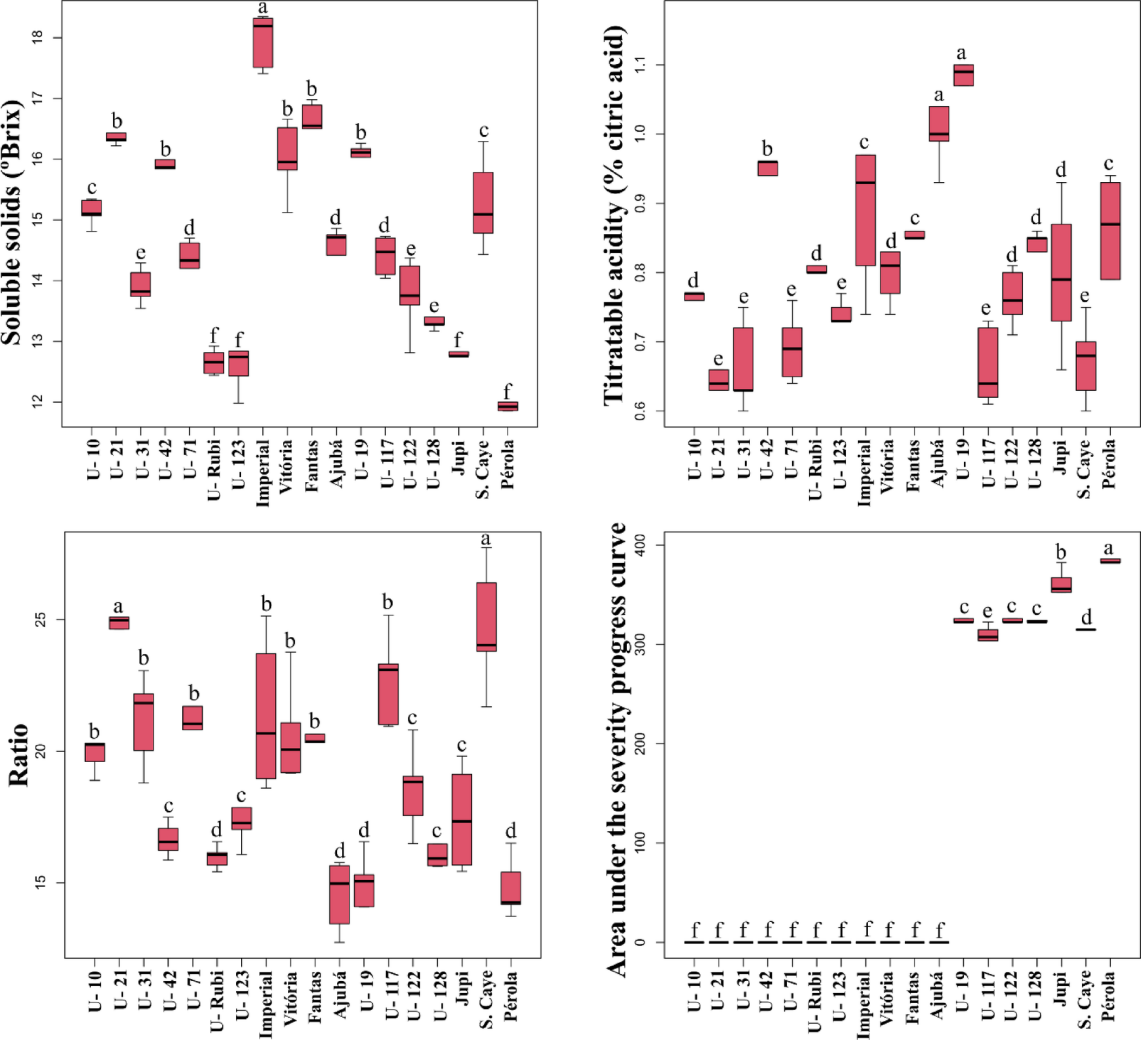
Boxplots for means of plant height (cm), D-leaf length (cm) number of seedlings, fruit weights with and without crown (respectively, in g), mean fruit diameter (cm), soluble solids (ºbrix), titratable acidity (% citric acid), SS-to-TA ratio (ratio), and area under the disease progress curve (AUDPC) to *Fusarium guttiforme* in 18 pineapple genotypes. The vertical line segments (whiskers) represent the minimum and maximum values. The black lines (—) identify the individual mean for each characteristic..

‘Unemat Rubi’ did not differ significantly from its male parent (‘Pérola’) in vegetative traits, with plant height of 100.94 cm, D-leaf length of 96.24 cm, and number of seedlings of 8.44 (Fig. 4). These traits are crucial for assessing plant growth^47^. Additionally, D-leaf length is an essential parameter for artificial flower induction, as it serves as a reliable reference for determining the appropriate timing for flower induction, mainly due to its positive correlation with infructescence weight and length at the harvesting stage^48^.

Fruit weights with and without crown (FMWC and FMWOC, respectively) ranged from 715.92 to 2145.11 g (FMWC) and 685.48 to 1994.89 g (FMWOC) (Fig. 4). ‘Unemat Rubi’ had mean values of 1495.67 g (FMWC) and 1364.47 g (FMWOC), categorizing its fruits as small to medium-sized (0.9 to 1.6 kg) according to Brazilian standards. These values were higher than those found for its parents (‘Pérola’ and ‘BRS Imperial’) and ‘Smooth Cayenne’, the most widely consumed pineapple cultivar the international market^3^. ‘Smooth Cayenne’ pineapples are within Class I (1.1 to 1.5 kg) for export purposes. Therefore, the superior weight of ‘Unemat Rubi’ pineapples compared to ‘Smooth Cayenne’ makes them suitable for both domestic and international markets.

Regarding chemical fruit traits, soluble solids ranged from 17.96 to 12.66 °Brix, which fall within the expected range for fresh fruit consumption (Fig. 4). Brazilian classification standards establish that a ripe pineapple ideal for consumption should have a minimum of 12°Brix^49^. ‘Unemat Rubi’ pineapples met this standard, exhibiting 12.66 °Brix. Titratable acidity (TA) values ranged from 0.66 to 1.03. TA is generally expressed as a percentage of citric acid, which varies from 0.32 to 1.22%^50^, depending on the cultivar, fruit ripening stage, climate factors, and plant mineral nutrition^51^. The mean TA of ‘Unemat Rubi’ pineapples was 0.80, which is within the limits established by Brazilian standards for both fresh consumption and processing.

Regarding response to fusariosis, 11 genotypes (four cultivars and seven clones), including ‘Unemat Rubi’, showed no symptoms following inoculation with *F. guttiforme*. The area under the disease progress curve (AUDPC) ranged from 0 to 386.25. Resistant genotypes exhibited no leaf lesions or internal stem infections 120 days after inoculation (DAI), obtaining a score of 0. However, susceptible genotypes exhibited leaf along with gummosis, resulting in plant death. The first symptoms appeared at 45 DAI in susceptible controls (cultivars ‘Pérola’ and ‘Jupi’) and at 60 DAI in clones 19, 117, 122, and 128. This confirms that the isolate mixture used was effective in inducing infection in seedlings of well-known susceptible cultivars used as control.

The climatic data recorded during the evaluation of fusariosis (Fig. 2) favoured the development of the disease, as mild temperatures and high humidity create ideal conditions for fungal growth, promoting the appearance of symptoms in the plants. These results are consistent with those of Lira Junior et al.^14^, who reported distinct responses to inoculation with *F. guttiforme* in the best 18 genotypes selected for fruit weight soluble solids. This variability in genotype responses to *F. guttiforme* can be attributed to genetic differences, as the plants were propagated by seeds obtained through hybridization.

Considering the essential criteria for a competitive cultivar in the international market, such as fruit weight, soluble solids, and pulp color, the choice should depend on specific uses and markets, adaptation to the growing environment, and the availability and quality of seedlings^52^. In this context, ‘Unemat Rubi’ stood out among the 11 evaluated pineapple clones by combining the highest number of desirable traits. Among genotypes with cylindrical fruits, yellow pulp, resistance to fusariosis, and the absence of leaf spines, ‘Unemat Rubi’ was superior in fruit weight and diameter than its parents (‘Pérola’ and ‘BRS Imperial’) and the other genotypes in Cluster I, which explains its selection. Furthermore. the number of cultivars possessing these traits available in the domestic market remains limited, despite Brazil’s vast size and diverse edaphoclimatic conditions across the country. The introduction of ‘Unemat Rubi’ into the market represents a significant advancement in pineapple breeding, addressing the demands of both the international market and local growers.

### Canonical variables

The canonical variables analysis, aligned with the Gower distance results, explained 96.4% of the total variance, highlighting AUDPC as the main factor distinguishing between resistant and susceptible genotypes to fusariosis, with no significant correlation between the physical and chemical characteristics of the fruit and resistance to the disease.

**Fig. 5:**
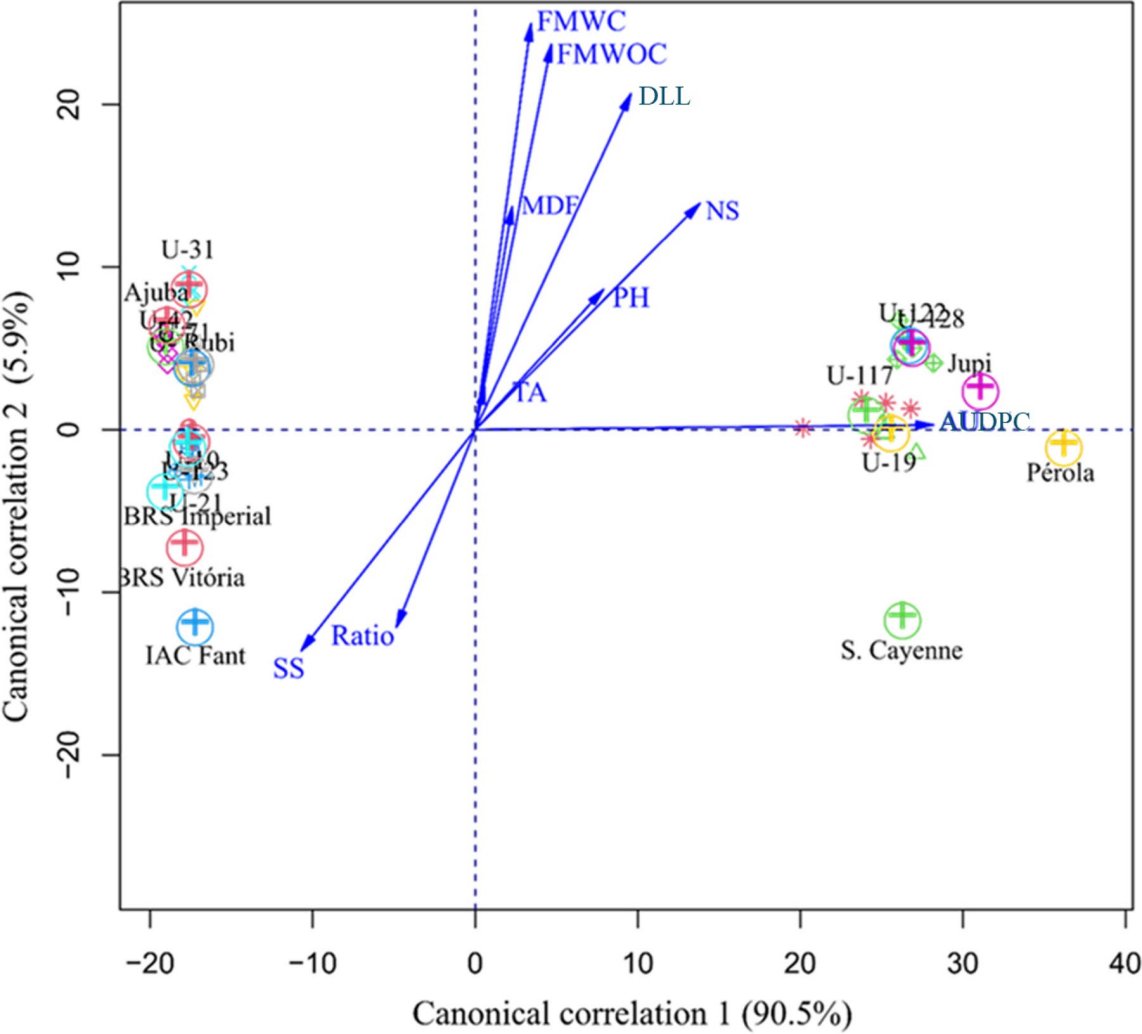
Scatter plot of scores of the first two canonical variables (Can1 and Can2) for plant height (PH in cm), D-leaf length (DLL in cm), number of seedlings (NS), fruit weights with and without crown (FMWC and FMWOC, respectively, in g), mean fruit diameter (MFD in cm), soluble solids (SS in ºbrix), titratable acidity (TA in % citric acid), SS-to-TA ratio (SS/TA), and area under the disease progress curve (AUDPC) to *Fusarium guttiforme* in 18 pineapple genotypes.

The joint analysis of the sets of variables revealed that the first two canonical variables explained the total variance, with Can1 explaining 90.5% and Can2 explaining 5.9%, enabling a more accurate interpretation through a two-dimensional plot (Fig. 5). AUDPC contributed the most to the genetic divergence in Can1 (1.03), allowing for the differentiation of genotypes into two clusters based on their response to fusariosis. FMWOC contributed the most in Can2 (7.47) (Fig. 5).

AUDPC is a key variable to quantify the genetic resistance of pineapple genotypes to fusariosis. Lower AUDPC values ​​indicate that certain genotypes possess resistance genes that reduce disease progression, making plants less susceptible to infection by *Fusarium guttiforme*. This resistance is mediated by specific defense genes, which activate effective mechanisms against the pathogen. In addition to the genetic response, plants can trigger additional biochemical and physiological defenses, such as: synthesis of antimicrobial compounds that inhibit fungal growth; structural reinforcement of the cell wall, with deposition of lignin and callose, hindering pathogen penetration; and hormonal regulation, involving salicylic acid and ethylene, which modulate immune responses and induce systemic resistance. These combined factors allow resistant plants to contain the infection more efficiently, which is reflected in the lower AUDPC observed at Unemat Rubi.

In addition to AUDPC, canonical variable 2, fruit mass with crown, highlights the variation in fruit size, a fundamental factor for the commercial viability of pineapple and differentiation of the genotypes studied. Since these variables are on separate axes, it indicates that there are cultivars that combine resistance with different fruit sizes, without a clear pattern between them. Therefore, there may be resistant genotypes but with small fruits, productive genotypes but susceptible to the disease, and genotypes that combine resistance and good productivity (the most commercially desirable), as is the case of Unemat Rubi.

According to the graphical analysis, no relationship was observed among the sets of fruit quality-related physical and chemical variables and resistance to fusariosis. However, the AUDPC vector extended toward the susceptible genotypes, which exhibited higher values for this trait. In contrast, the resistant genotypes, including ‘Unemat Rubi’, were positioned on the opposite side of the graph with an AUDPC score of 0. The canonical variable analysis was consistent with the results from the Gower distance-based dendrogram (Fig. 3); however, it provided canonical correlation coefficients, which indicate the strength and direction of relationships among the variable sets, identifying those with the highest canonical loadings and the greatest influence on the linear combinations under analysis^53^.

AUDPC did not correlate with other variables because disease resistance appears to be independent of morphological and productive traits. This can be advantageous for genetic improvement, as it means that cultivars can be developed that are both productive and resistant simultaneously.

The consistency of results across multiple analytical approaches strengthens the validity of the findings. This consistency suggests reliability of the results, as they did not depend on a single analytical methodology but were validated through multiple approaches. Thus, the convergence of the results reinforces the validity of the conclusions.

### Correlation networks

**Fig. 6:**
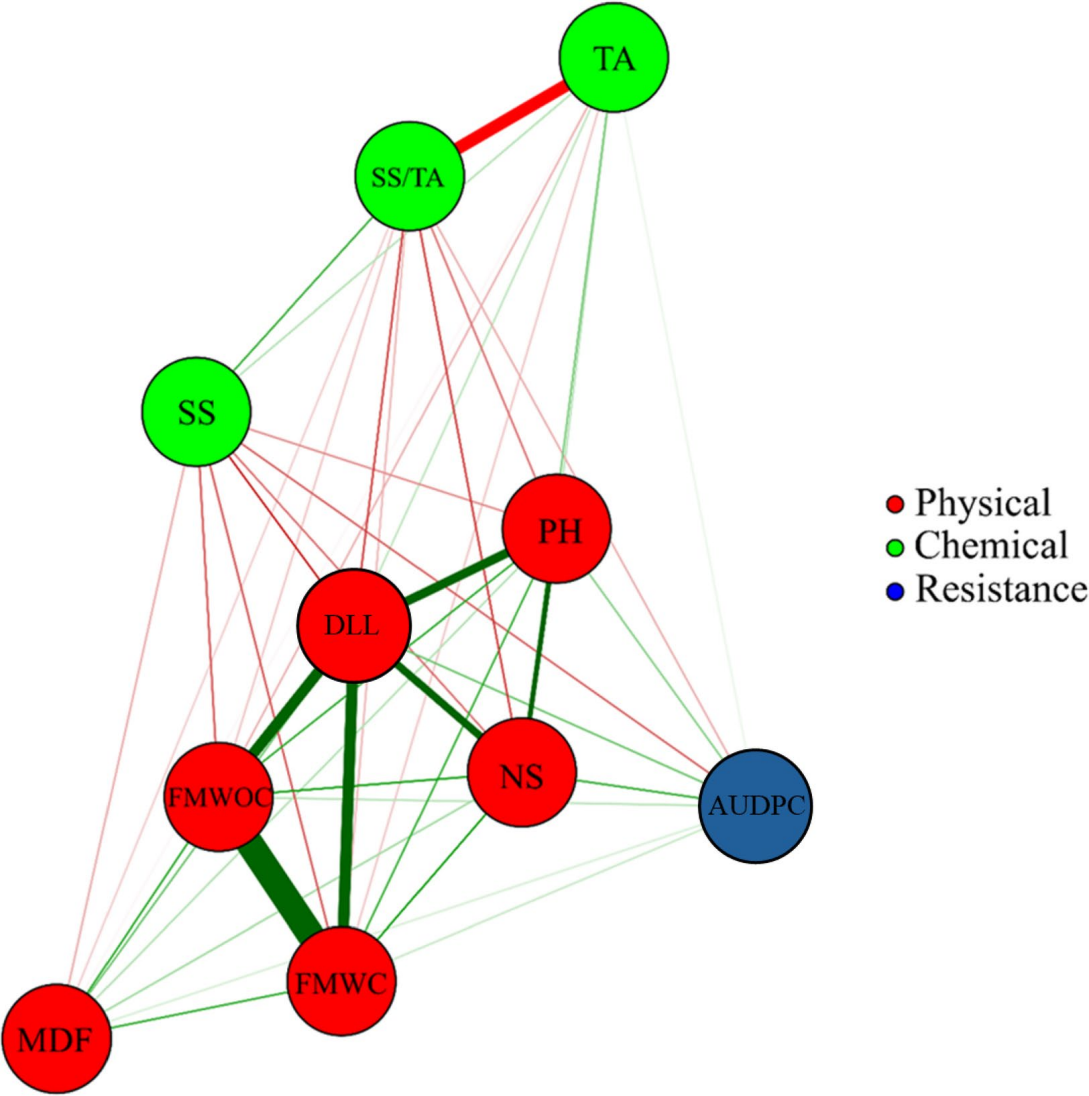
Correlation network for physical and chemical fruit traits and resistance to fusariosis in 18 pineapple genotypes. Red lines represent negative correlations and green lines represent positive correlations. The thickness of the line is proportional to the magnitude of the correlation. The highlighted lines represent correlation coefficients greater than 0.6 in absolute value. Physical variables: plant height (PH in cm), D-leaf length (DLL in cm), number of seedlings (NS), fruit weights with and without crown (FMWC and FMWOC, respectively, in g), and mean fruit diameter (MFD in cm). Chemical variables: soluble solids (SS in ºbrix), titratable acidity (TA in % citric acid), and SS-to-TA ratio Ratio (SS/TA). Response of plants to infection by *Fusarium guttiforme*: area under the disease progress curve (AUDPC).

The network correlation constructed with the sets of physical and chemical fruit traits and fusariosis resistance denotes the formation of a main cluster consisted of physical fruit variables, while chemical fruit variables and fusariosis resistance were positioned on the opposite side of the network (Fig. 6).

Correlations were observed within, but not between, physical and chemical fruit variables. AUDPC did not correlate with any other variable, suggesting that fusariosis resistance is not associated with physical and chemical traits of fruits. Regarding physical fruit variables, a high positive correlation (0.99) was found between FMWC and FMWOC, as expected. Similarly, a correlation of approximately 1.0 was reported between these two variables, indicating that there is no need to evaluate both characteristics, thus simplifying the process^54^. D-leaf length (DLL) was strongly and significantly correlated with FMWC and FMWOC, both with a value of 0.74. This result contrasts with those reported by Kuster et al.^55^, who assessed DLL at the flower induction stage and found weak and very weak correlations with fruit weight with and without crown. However, D-leaf length is considered a reference for determining the appropriate timing for flower induction, as it is typically positively correlated with infructescence weight and length at the harvesting stage^48^. Therefore, more vigorous pineapple plants tend to produce heavier fruits^56,57^.

The correlation network was primarily constructed to identify significant relationships between fruit quality variables, which are faster and easier to measure, and fusariosis resistance, which is more time-consuming to assess. However, this relationship was not confirmed (Fig. 6).

### Variance component estimates using the method of moments

The estimation of variance components revealed that most of the phenotypic variance for the majority of the evaluated traits is attributed to the genetic component (Table 3). This was evidenced by high coefficients of genetic variation and variation indices greater than 1.0, except for mean fruit diameter (MFD), suggesting genetic contribution to the differences observed among the evaluated phenotypes.

This factor is even more significant considering the crop’s genetic structure, as this variation is fully inherited in asexually propagated plants, such as pineapple. Consequently, the environmental variance for these traits showed a lower range, indicating a reduced environmental effect on these traits based on the population type and the estimation method used.

**Table 3:** Estimates of variance components in 18 pineapple genotypes (10 clones and 8 cultivars) based on 10 traits: plant height (PH in cm), D-leaf length (DLL in cm), number of seedlings (NS), fruit weights with and without crown (FMWC and FMWOC, respectively, in g), mean fruit diameter (MFD in cm), soluble solids (SS in ºbrix), titratable acidity (TA in % citric acid), SS-to-TA ratio (SS/TA), and area under the disease progress curve (AUDPC) to *Fusarium guttiforme.*

Parameters	PH	DLL	NS	FMWC	FMWOC	MFD	TSS	TA	SS/TA	AUDPC
Phenotypic variance	123.87	151.21	7.78	18,8371.00	167,800.70	1.39	2.61	0.02	10.80	28,351.57
Genetic variance	111.74	146.43	7.38	185,646.10	164,989.40	1.09	2.56	0.01	10.19	28,339.93
Environmental variance	12.12	4.78	0.41	2724.84	2811.23	0.30	0.05	0.00	0.61	11.64
Broad-sense heritability	90.21	96.84	94.78	98.55	98.32	78.76	98.23	94.58	94.34	99.96
Coefficient of genotypic variation	11.09	13.77	39.08	30.47	30.95	8.99	10.93	15.03	16.65	129.60
Variation index	1.36	2.48	1.91	3.69	3.43	0.86	3.33	1.87	1.83	22.06
Accuracy	0.95	0.98	0.97	0.99	0.99	0.89	0.99	0.97	0.97	0.99
Overall mean	95.30	87.88	6.95	1414.14	1312.42	11.63	14.64	0.81	19.17	129.90
Coefficient of variation	8.16	5.56	20.49	8.25	9.03	10.44	3.28	8.04	9.11	5.87

Broad-sense heritability estimates exceeded 90% for most traits, ranging from 78.76 to 99.96% for MFD and AUDPC, respectively. These results indicate that genetic factors accounted for more than 90% of the total phenotypic variance in fruit quality traits (except MFD) and fusariosis resistance, highlighting the potential for genetic progress through selection.

This is particularly significant for the UNEMAT pineapple breeding program, as the selected clones transmitted all favorable genetic variation to their descendants. In contrast, the environmental effect on MFD was greater than that on the other traits, resulting in lower heritability and accuracy values.

Similarly, Abreu et al.^54^, reported estimates of approximately 1.0 for resistance to fusariosis when evaluating 19 pineapple clones using the REML/BLUP methodology, attributing 99% of the total variance to inheritable genetic effects. The convergence of these results minimizes the likelihood that the observed values are due to error or bias specific to a statistical method. This finding reinforces the hypothesis that resistance to fusariosis can be controlled by one or a few genes, as suggested in previous studies^58,59^. Nonetheless, more comprehensive studies evaluating larger populations under appropriate experimental designs are necessary to confirm this hypothesis.

Coefficients of variation (CV) were 3.28 and 20.49 for soluble solids (SS) and number of seedling (NS), respectively. The classification range for CV was based on the methodology proposed by Garcia^60^, which categorizes CV values using the mean, standard deviation, and a normal distribution of the variable.

In this context, despite the higher environmental variance, the CV value for MFD (10.44) was lower than that for NS (20.49), as NS did not follow a normal distribution. Additionally, the CV values were estimated based on residuals rather than environmental variance; therefore, MFD showed lower accuracy and CV values compared to NS.

Several studies evaluating pineapple plants have indicated that the contribution of genetic and environmental variances varies depending on the population under study and is commonly observed in traits such as PH, FMWC, FMWOC, TSS, and TA^54,61^.

Variations in sample size, environmental conditions, genetic population structure, experimental design, and estimation methods can explain the different ranges of variance affecting genetic parameter values. This suggests that these estimates are population-specific and cannot be generalized. Variance component estimates and genetic parameter data for a plant population are crucial for understanding the genetic control of traits under selection, enabling the adoption of optimal breeding strategies.

When comparing the multivariate analyses used in this study, PCA indicated that the variables AACPS and MF were the components responsible for explaining most of the data variability. The Gower distance formed two clusters, separating Fusarium-resistant and Fusarium-susceptible individuals, while the correlation network did not reveal significant relationships between the physical and chemical characteristics of the fruit and *Fusarium* resistance.

These findings of this study provide valuable information for pineapple breeding programs focused on improving fruit quality and resistance to fusariosis. The cultivar ‘Unemat Rubi’, registered at the Brazilian Ministry of Agriculture and Livestock under number 56,622, marks a significant advancement in pineapple breeding by combining superior fruit quality with disease resistance.

These attributes make it a promising candidate for commercial expansion, with the potential to support sustainable high-yield pineapple production, benefiting both growers and consumers. However, further research is essential to assess the performance of ‘Unemat Rubi’ across diverse pineapple production centers, validating its adaptability to varying agroclimatic conditions.

## Conclusions

Among pineapple genotypes combining resistance to fusariosis, cylindrical fruit shape, yellow pulp, and absence of leaf spines, ‘Unemat Rubi’ showed superior fruit weight and diameter compared to its female parent ‘BRS Imperial’. These attributes make ‘Unemat Rubi’ a promising cultivar for commercial expansion, particularly in the international market, offering a sustainable high-yield crop alternative with the potential to increase fruit weight by 500 g.

Multivariate analyses using Gower distance and canonical variables proved convergent and effective in identifying superior clones, highlighting ‘Unemat Rubi’ for combining desirable traits related to plant morphology, fruit quality, and resistance to fusariosis. Furthermore, correlation networks revealed no significant relationships between physical and chemical fruit traits and resistance to fusariosis.

The pineapple cultivar Unemat Rubi is being maintained and propagated under field conditions as well as through micropropagation, with a focus on distributing planting material to pineapple growers for multi-environmental trials to assess its stability and adaptability in different agro-climatic conditions. In addition, genomic tools such as molecular markers will be used to ensure the genetic identity of the cultivar.

## Electronic supplementary material

Below is the link to the electronic supplementary material.

Supplementary Material 1

## Data Availability

Data is provided within the manuscript and supplementary information files.
